# Full-term newborns with normal birth weight requiring special care in a resource-constrained setting

**DOI:** 10.11604/pamj.2013.15.36.576

**Published:** 2013-05-29

**Authors:** Bolajoko O. Olusanya

**Affiliations:** 1 Maternal and Child Health, Reproductive Health Unit , Department of Community Health and Primary Care, College of Medicine, University of Lagos, Surulere, Lagos, Nigeria

**Keywords:** antenatal care, developing country, emergency cesarean delivery, hyperbilirubinemia, neonatal intensive care, special baby care

## Abstract

**Introduction:**

The level of clinical care and facilities to support the often more viable full-term newborns with normal birth weight compared with preterm/low birth weight newborns that require special care at birth are likely to be attainable in many resource-poor settings. However, the nature of the required care is not evident in current literature. This study therefore set out to determine maternal and perinatal profile of surviving full-term newborns with normal birth weight in a poorly-resourced setting.

**Methods:**

A retrospective cohort study of newborns with gestational age ≥37 weeks and birth weight ≥2500g recruited in an inner-city maternity hospital in Lagos, Nigeria. Primary factors/outcomes were determined by multivariate logistic regression analyses and population attributable risk (PAR).

**Results:**

Of the 2687 full-term newborns with normal birth weight studied, 242 (9.0%) were admitted into special care baby unit (SCBU) representing 53.6% of all SCBU admissions. Fetal distress, low 5-minute Apgar scores, neonatal sepsis and hyperbilirubinemia as well as maternal factors such as primiparity, type of employment, lack of antenatal care and emergency cesarean delivery were predictive of SCBU admission. The leading contributors to SCBU admission were neonatal sepsis (PAR=96.8%), and hyperbilirubinemia (PAR=58.7%).

**Conclusion:**

A significant proportion of newborns requiring special care are full-term with normal birth weight and are associated with modifiable risk factors that can be effectively addressed at appropriately equipped secondary-level hospitals. Prenatal maternal education on avoidable risk factors is warranted.

## Introduction

Of the estimated 123 million babies born annually in the developing world, the vast majority (84% or more) are full-term (≥37 completed weeks of gestation) [1, 2]. While all infants have immature immune system at birth, full-term compared with preterm infants are biologically less susceptible to adverse birth outcomes as they usually have most elements of a mature immune system with additional protection through passive immunity from transplacental transfer of antibodies which enhance their ability to survive and thrive well into adulthood [3]. Similarly, infants with birth weight of at least 2500g are 20 to 40 times more likely to survive or many times less likely to have severe morbidity and disabilities compared to low birth weight [4, 5]. Notwithstanding, full-term infants (the vast majority of whom will have normal birth weight) still constitute a significant proportion (up to 54%) of newborns requiring intensive or special care primarily as a result of intrapartum complications, infections, birth asphyxia as well as sub-optimal obstetric care [6–8].

Increasing but limited number of studies have shown that full-term graduates of neonatal intensive care units (NICU)/special care baby unit (SCBU) are at increased risk of a range of adverse short and long-term outcomes including high rates of post-neonatal rehospitalization [7, 9–12]. Mothers of full-term infants admitted into NICU/SCBU are also likely to face more emotional trauma and anxiety with consequences for maternal bonding compared with mothers of preterm and low birth weight infants who are more likely to appreciate and accept the need for special care for their babies [13]. The knowledge of the factors associated with the admission of such infants into NICU/SCBU should be helpful for their anticipatory management in low-income countries [7, 8]. The constraints imposed by the widespread lack of NICU facilities required more frequently by preterm and low birth weight newborns have also prompted suggestions for a higher priority in providing effective services for the more viable full-term newborns requiring special care in order to optimize the limited human and financial resources in these countries [14, 15]. This retrospective study therefore set out to determine the maternal and perinatal profile of full-term newborns that are likely to graduate from NICU/SCBU in a resource-poor setting to facilitate improved services and better outcomes for such infants.

## Methods

### Study design and population

This retrospective cohort study was conducted at the Island Maternity Hospital (IMH) in Lagos, Nigeria among newborns consecutively enrolled between June 2005 and May 2007 under a previously reported universal newborn hearing screening (UNHS) program [16]. IMH is owned and managed by the state government as a public health institution. It is the oldest maternity hospital in Nigeria providing specialist services to several private and public hospitals within metropolitan Lagos. At the time of this study, the hospital had 180 beds for maternity services and a 15-bed SCBU equipped with incubators, stand-alone resuscitation units, suction machines, oxygen concentrators, infusion sets for intravenous fluids (not parenteral nutrition) and phototherapy units. Babies requiring intensive care including exchange blood transfusion were usually referred to a nearby children's hospital after a brief stay in SCBU. The study was conducted according to the guidelines laid down in the Declaration of Helsinki and all procedures involving human subjects/patients were approved by Lagos State Health Management Board, Nigeria (Ref. No: SHMB/728T dated 17^th^ December 2003) and University College London, UK (Ref. No: 03AM04 dated 9^th^ January 2004). Informed consent was obtained from all participating mothers in writing or thumb printing.

### Case definition

The target population consisted of all surviving full-term newborns during the study period with gestational age of at least 37 completed weeks and birth weight of at least 2500g. In the absence of any serious complications, this gestational/weight threshold is universally viable with minimal care. Thus, all preterm or low birth weight infants as well as those born full-term with low birth weight were excluded because of their higher risk of complications as earlier noted [2–5]. Combining birth weight with gestational age helped to minimize the impact of gestational age estimation based solely on maternal recall of last menstrual period (LMP).

### Study variables

The selected variables were guided by evidence from the literature, available data from the hospital and non-clinical data that could be reliably elicited from the mothers [6–12, 17]. They were grouped under maternal socio-demographic and obstetric factors as well as infant characteristics. Socio-demographic factors included maternal age, marital status, ethnicity, religion, education, occupation, social class and quality of residential accommodation (shared sanitation facilities or self-contained) [9, 10, 12]. Social classes were determined based on mother's education and father's occupation as validated in the study population [18]. Social class I was termed as “high”, II or III as “middle” and IV or V as “low”. Obstetric factors included parity, antenatal care, use of herbal drug in pregnancy, maternal HIV status, previous cesarean section, hypertensive disorders (inclusive of pre-eclampsia, eclampsia and pregnancy induced hypertension), antepartum hemorrhage, cephalopelvic disproportion, premature rupture of membranes, placenta previa, prolonged and/or obstructed labor, malpresentation (all non-cephalic presentations) and mode of delivery [6, 10, 12]. Neonatal characteristics included gender, gestational type, cord accidents, fetal distress, birth asphyxia (indexed by low Apgar scores at one minute and five minutes), sepsis (used collectively for septicemia, meningitis and pneumonia), hyperbilirubinemia and congenital defects [6–12, 17].

### Statistical analysis

Differences in the characteristics of babies by nursery type were first determined by two-tailed chi-square test in univariate analyses based on the odds ratios (OR) and the corresponding 95% confidence intervals (CI). Two separate logistic models were built for maternal factors and infant characteristics to reflect the hierarchical nature of these two sets of factors [19]. The first logistic model consisted of all maternal factors significantly associated (p*R* ^*2*^ statistic (a measure of explained variation in the model). The population attributable risk percentage (PAR) for each risk factor in the final model was estimated using the formula PAR= Pe(RR-1)/RR) where (Pe) was the proportion of the cases (SCBU babies) exposed to the risk factor and (RR) was the relative risk estimated by the adjusted odds ratio for each factor [20]. All statistical analyses were done with SPSS for Windows version 16.0 (SPSS Inc, Chicago, IL, USA).

## Results

A total of 3754 infants with mean gestational age of 37.8 weeks (SD: ±2.1 weeks) were enrolled under the primary study period out of which 8 infants with unknown birth weight were excluded. A further 1059 (28.2%) comprising 846 preterm infants and 213 term infants with low birth weight infants were excluded, 846 (79.9%) of whom were in WBN. Thus 2687 newborns (mean gestational age 38.6±1.2 weeks and mean birth weight 3.4±0.5kg) delivered by 2641 mothers met our inclusion criteria of full-term infants with normal birth weight out of which 242 (9.0%) were admitted into SCBU representing 53.6% of all (455) SCBU admissions. Median age at enrolment was 1 day (interquartile range: 1 to 2 days). Majority of the mothers were between the ages of 20-35 years, married, of the Yoruba ethnic group, professed Christianity, had a minimum of secondary education, were either self-employed or in regular employment and belonged to the middle social class (Table 1). The obstetric profile of the mothers is presented in Table 2. Slightly over half (52.6%) of the mothers were primiparous while 95 (3.6%) were grand multiparous (data not shown). Almost a third (31.2%) did not attend antenatal clinics for their current delivery and slightly over half (54.7%) of this group were primiparous. However, the difference in antenatal care utilization was not statistically significant by parity (p=0.143). About two-fifths (41.7%) delivered by cesarean section with emergency cesarean accounting for 78.2% in this group. HIV was confirmed in 5.5% of the mothers. Over half (52%) of the infants were male and only 0.5% had congenital anomalies (Table 3). The vast majority (97%) of newborns with sepsis and 62% of infants with hyperbilirubinemia were admitted into SCBU.

**Table 1:** Socio-demographic factors associated with neonatal admission into special care unit in an inner-city maternity hospital, Lagos, Nigeria

Factors	All mothers (%) n=2641		Mothers with babies in SCBU (%)¶ n=236	Mothers with babies in WBN (%)¶ n=2405	Unadjusted odds ratio (95% confidence interval)
**Age (Years) [a]**	< 20	40 (1.5)	4 (10.0)	36 (90.0)	1.12 (0.39 – 3.17)
	20 – 35	2289 (86.8)	207 (9.0)	2082 (91.0)	1.0
	> 35	308 (11.7)	24 (7.8)	284 (92.2)	0.85 (0.55 – 1.32)
**Marital status**	Married	2591 (98.1)	230 (8.9)	2361 (91.1)	1.0
	Not married	50 (1.9)	6 (12.0)	44 (88.0)	1.40 (0.59 – 3.32)
**Ethnicity**	Yoruba	2033 (77.0)	173 (8.5)	1860 (91.5)	1.0
	Hausa	65 (2.5)	4 (6.2)	61 (93.8)	0.71 (0.25 – 1.96)
	Ibo & Others	543 (20.6)	59 (10.9)	484 (89.1)	1.31 (0.96 – 1.79)
**Religion**	Muslim	1045 (39.6)	86 (8.2)	959 (91.8)	1.0
	Christianity	1596 (60.4)	150 (9.4)	1446 (90.6)	1.16 (0.88 – 1.53)
**Education**	None	53 (2.0)	4 (7.5)	49 (92.6)	1.0
	Primary/Secondary	1490 (56.4)	141 (9.5)	1349 (90.5)	1.28 (0.45 – 3.60)
	Tertiary	1098 (41.6)	91 (8.3)	1007 (91.6)	1.11 (0.39 – 3.14)
**Occupation**	None	558 (21.1)	35 (6.3)	523 (93.7)	1.0
	Self-employment	1249 (47.3)	129 (10.3)	1120 (89.7)	1.72 (1.17 – 2.54)**
	Regular employment	834 (31.6)	72 (8.6)	762 (91.4)	1.41 (0.93 – 2.15)
**Social class**	High	513 (19.4)	41 (8.0)	472 (92.0)	1.0
	Middle	1787 (67.7)	157 (8.8)	1630 (91.2)	1.11 (0.76 – 1.59)
	Low	341 (12.9)	38 (11.1)	303 (88.9)	1.44 (0.91 – 2.30)
**Housing sanitation facilities**	Not Shared	1429 (54.1)	109 (7.6)	1320 (92.4)	1.0
	Shared	1212 (45.9)	127 (10.5)	1085 (89.5)	1.42 (1.08 – 1.85)*

¶ Row percentage Missing data: [a]=4 (0.2%)

* p < 0.05

** p < 0.01

SCBU=special care baby unit; WBN=well baby nursery

**Table 2:** Obstetric factors associated with neonatal admission into special care unit in an inner-city maternity hospital, Lagos, Nigeria

Factors		All mothers (%) n=2641	Mothers with babies in SCBU (%)¶ n=236	Mothers with babies in WBN (%)¶ n=2405	Unadjusted odds ratio (95% confidence interval)
**Primiparity**	No	1253 (47.4)	87 (6.9)	1166 (93.1)	1.0
	Yes	1388 (52.6)	149 (10.7)	1239 (89.2)	1.61 (1.22 – 2.13)**
**Lack antenatal care**	No	1816 (68.8)	104 (5.7)	1712 (94.3)	1.0
	Yes	825 (31.2)	132 (16.0)	693 (84.0)	3.14 (2.39 – 4.11)***
**Herbal drug use in pregnancy**	No	2111 (79.9)	192 (9.1)	1919 (90.9)	1.0
	Yes	530 (20.1)	44 (8.3)	486 (91.7)	0.91 (0.64 – 1.27)
**Maternal HIV**	No	2497 (94.5)	226 (9.1)	2271 (90.9)	1.0
	Yes	144 (5.5)	10 (6.9)	134 (93.1)	0.75 (0.39 – 1.45)
**Previous cesarean section**	No	2369 (89.7)	213 (9.0)	2156 (91.0)	1.0
	Yes	272 (10.3)	23 (8.5)	249 (91.5)	0.94 (0.60 – 1.47)
**Hypertensive disorders**	No	2531 (95.8)	219 (8.7)	2312 (91.3)	1.0
	Yes	110 (4.2)	17 (15.5)	93 (84.5)	1.93 (1.13 – 3.30)*
**Ante-partum hemorrhage**	No	2622 (99.3)	232 (8.9)	2390 (91.2)	1.0
	Yes	19 (0.7)	4 (21.1)	15 (78.9)	2.75 (0.90 – 8.35)
**Cephalopelvic disproportion**	No	2517 (95.3)	216 (8.6)	2301 (91.4)	1.0
	Yes	124 (4.7)	20 (16.1)	104 (83.9)	2.05 (1.24 – 3.37)**
**Premature rupture of membranes**	No	2625 (99.4)	233 (8.9)	2392 (91.1)	1.0
	Yes	16 (0.6)	3 (18.8)	13 (81.2)	2.37 (0.67 – 8.37)
**Placenta previa**	No	2610 (98.8)	232 (8.9)	2378 (91.1)	1.0
	Yes	31 (1.2)	4 (12.9)	27 (87.1)	1.51 (0.53 – 4.38)
**Prolonged/obstructed labor**	No	2351 (89.4)	174 (7.4)	2177 (92.6)	1.0
	Yes	290 (10.6)	62 (21.4)	228 (78.6)	3.40 (2.47 – 4.67)***
**Malpresentation**	No	2506 (94.9)	216 (8.6)	2290 (91.4)	1.0
	Yes	135 (5.1)	20 (14.8)	115 (85.2)	1.84 (1.12 – 3.02)*
**Mode of delivery**	Elective cesarean	240 (9.1)	15 (6.3)	225 (93.8)	1.0
	Emergency cesarean	860 (32.6)	150 (17.4)	710 (82.6)	3.17 (1.83 – 5.50)***
	Spontaneous vertex	1541 (58.3)	71 (4.7)	1470 (95.4)	0.72 (0.41 – 1.29)

¶ Row percentage

* p < 0.05

** p < 0.01

*** p < 0.001

SCBU=special care baby unit; WBN=well baby nursery

**Table 3:** Profile of full-term neonates admitted into special care unit in an inner-city maternity hospital, Lagos, Nigeria

Profile		All newborns (%) n=2687	Admitted into SCBU (%)¶ n=242	Admitted into WBN (%)¶ n=2445	Unadjusted odds ratio (95% confidence interval)
**Sex**	Female	1290 (48.0)	115 (9.0)	1175 (91.1)	1.0
	Male	1397 (52.0)	127 (9.1)	1270 (90.9)	1.02 (0.78 – 1.33)
**Gestational type**	Singletons	2581 (96.0)	227 (8.8)	2354 (91.2)	1.0
	Twins/Triplets	106 (4.0)	15 (14.2)	91 (85.8)	1.71 (0.97 – 3.00)
**Cord accidents**	No	2672 (99.4)	240 (9.0)	2432 (91.0)	1.0
	Yes	15 (0.6)	2 (13.3)	13 (86.7)	1.56 (0.35 – 6.95)
**Fetal distress**	No	2581 (96.1)	211 (8.2)	2370 (91.8)	1.0
	Yes	106 (3.9)	31 (29.2)	75 (70.8)	4.64 (2.99 – 7.22)***
**1-minute Apgar score at [a]**	7 – 10	291 (10.9)	8 (2.7)	283 (97.3)	1.0
	0 - 6	2372 (89.1)	234 (9.9)	2138 (90.1)	3.87 (1.89 – 7.92)***
**5-minute Apgar score at [b]**	7 – 10	2000 (75.1)	109 (5.4)	1891 (94.6)	1.0
	0 - 6	663 (24.9)	133 (20.1)	530 (79.9)	4.35 (3.32 – 5.71)***
**Neonatal sepsis**	No	2621 (97.5)	178 (6.8)	2443 (93.2)	1.0
	Yes	66 (2.5)	64 (97.0)	2 (3.0)	439.19 (106.63 – 1809.00)***
**Hyperbilirubinemia**	No	2637 (98.1)	211 (8.0)	2426 (92.0)	1.0
	Yes	50 (1.9)	31 (62.0)	19 (38.0)	18.76 (10.42 – 33.78)***
**Congenital defects**	No	2674 (99.5)	241 (9.1)	2433 (91.0)	1.0
	Yes	13 (0.5)	1 (7.7)	12 (92.3)	0.84 (0.11 – 6.50)

¶ Row percentage Missing data: [a],[b]=24 (0.9%)

*p < 0.05; ** p < 0.01

*** p < 0.001; SCBU=special care baby unit; WBN=well baby nursery

In the univariate analyses of maternal factors, occupation, sanitation facilities in residence, parity, antenatal care, hypertensive disorders, cephalopelvic disproportion, prolonged/obstructed labor, malpresentation and mode of delivery were significantly associated with SCBU admission. Infant factors significantly associated with SCBU admission were fetal distress, low Apgar scores at 1 and 5 minutes, neonatal sepsis and hyperbilirubinemia. Prolonged/obstructed labor showed significant interaction with mode of delivery (pTable 4. Mothers of newborns admitted into SCBU were significantly likely to be primiparous (OR:1.56; 95% CI:1.10-2.21), self-employed (OR:2.63; 95% CI:1.57-4.40) or in regular employment (OR:1.82; 95% CI:1.05-3.16), lacking antenatal care (OR:2.00; 95% CI:1.41-2.82) and delivered by emergency cesarean section (OR:2.39; 95% CI:1.12-5.08).

**Table 4:** Predictors of SCBU admission among full-term neonates and the corresponding population attributable risk

Risk factor*	¶Adjusted odds ratio (95% confidence interval)	p-value	Population attributable risk percentage
**Maternal factors**			
**Occupation**			
Self employment	2.63 (1.57 – 4.40)	<0.001	6.4
Regular employment	1.82 (1.05 – 3.16)	0.035	3.9
Primiparity	1.56 (1.10 – 2.21)	0.012	3.8
Lack of antenatal care	2.00 (1.41 – 2.82)	<0.001	8.0
Prolonged/obstructed labor§	4.75 (0.85 – 26.58)	0.076	-
Emergency cesarean delivery	2.39 (1.12 – 5.08)	0.024	10.1
**Infant factors**			
Fetal distress	2.79 (1.57 – 4.81)	<0.001	18.7
5-minute Apgar score <7	3.23 (2.30 – 4.55)	<0.001	13.9
Neonatal sepsis	486.50 (112.09 – 2111.52)	<0.001	96.8
Hyperbilirubinemia	18.62 (8.82 – 39.32)	<0.001	58.7

Reference category as shown in Tables 1–3.

¶ Adjusted for all covariates, Apgar scores at 1 minute and interaction terms between prolonged obstructed labor and mode of delivery.

§ Interaction of mode of delivery and prolonged/obstructed labor in the final model (p=0.035); Hosmer-Lemeshow test: p=0.505; Pseudo-*R* ^2^=0.415; c-statistic=0.875 (p < 0.001)

Prolonged/obstructed labor significantly (p=0.035) modified the effect of mode of delivery on SCBU admission. Newborns delivered by emergency cesarean section associated with prolonged/obstructed labor had 2.69-fold increased risk of SCBU admission compared to newborns delivered by elective cesarean section without prolonged/obstructed labor. Neonatal sepsis (OR:486.60; 95% CI:112.09-2111.52) and hyperbilirubinemia (OR:18.62; 95% CI:8.82-39.32) emerged as the factors with the largest odds. Fetal distress (OR:2.79; 95% CI:1.57-4.55) and low five-minute Apgar scores (OR:3.23; 95% CI:2.30-4.55) were also predictive of SCBU admission.

The final model showed satisfactory discriminatory powers (c-statistic=0.875) and the factors explained about 42% variation in SCBU admission in this population (Nagelkerke Pseudo--*R* ^*2*^=0.415). There was no evidence of a lack of model fit (Hosmer-Lemeshow test: p=0.505). The leading contributors to SCBU admission were neonatal sepsis (PAR=96.8%) and hyperbilirubinemia (PAR=58.7%). Since PAR was calculated separately for each risk factor, their sum was not constrained to 1 (100%).

## Discussion

Barring the lack of data on early neonatal deaths, this study has shown that full-term/viable newborns constitute a significant proportion of newborns that require special care after birth consistent with findings from other studies [6–8, 10]. This study has also highlighted the potential impact of maternal health-seeking behavior underpinned by factors such as occupation, lack of antenatal care and emergency cesarean section on adverse perinatal outcomes which portend significant long-term sequelae for full-term survivors but can be effectively managed at properly equipped secondary-level hospitals.

The findings on early-onset sepsis, hyperbilirubinemia and low five-minute Apgar scores (used as proxy for birth asphyxia [17]) are consistent with several studies that have established these conditions as leading determinants of SCBU admission and risk factors for early neonatal deaths [8, 21, 22]. As with most studies in developing countries, septicemia, pneumonia and meningitis were collectively described as neonatal sepsis because of the similarity in their causative organisms and clinical presentation [23]. Gentamicin was the first-line antimicrobial therapy in this hospital due to the gram negative organisms commonly associated with sepsis. The drug is not only known to be effective but it is perhaps one of the most affordable in resource-poor countries but requires close monitoring and blood level measurements because of its ototoxic effects.

Hyperbilirubinemia is perhaps the commonest illness requiring (re)hospitalization soon after birth worldwide. Determination and close monitoring of the serum total bilirubin levels as well as timely initiation of phototherapy when required are effective in managing this condition in the vast majority of the affected infants. However, it was not uncommon for physicians and nurses to rely on visual estimation of the severity of jaundice because of the lack of facilities for effective monitoring of bilirubin levels. Like in other resource-poor settings, simple tools for predicting infants who are likely to have severe hyperbilirubinemia such as hand-held icterometer or portable BiliCheck device for transcutaneous bilirubin measurements were lacking. It is therefore necessary to educate parents to recognize and promptly respond to early signs of severe hyperbilirubinemia more so as the onset of jaundice in majority of such cases occur following hospital discharge.

Similarly, birth asphyxia could not be diagnosed routinely which prompts the widespread use of surrogates like Apgar scores. However, studies have shown that low five-minute Apgar scores (17,24-26]. Timely and on-going comprehensive neurologic assessment is therefore warranted for graduates of SCBU with a history of birth asphyxia. The proportion of infants with respiratory distress could not be ascertained but improved resuscitation practices should be helpful in reducing the effects of this risk factor.

Evidence from this study would suggest that the potential impact of the maternal factors on perinatal outcomes could be moderated. For example, the increased risk associated with full-time/regular employment may not be unrelated to the common practice among working women to delay the commencement of their statutory three months maternity leave as close to the delivery period as possible in order to spend longer period with their baby after delivery regardless of the considerable stress of living and working in a busy commercial city where this study was conducted. Similarly, self-employed women usually engaged in small trading were likely to be driven by economic reasons to continue with their business regardless of the stress and unaware of the potential risks to the unborn child. Lack of antenatal care is associated with several adverse perinatal outcomes in developing countries especially among multiparous mothers who often may consider such services as unnecessary based on past delivery experiences [27, 28]. It was therefore not surprising to find such a high rate of emergency cesarean delivery and the associated risk of SCBU admission which accords with an earlier observation in this population [28]. In fact, over half of the mothers who delivered by emergency cesarean section had no antenatal care. The lack of antenatal care in almost a third of primiparous mothers in this population also merits further investigation for appropriate intervention. It was also not uncommon for some mothers who had received antenatal care to end-up with emergency cesarean section if they had cultural aversion to a probable surgical intervention and/or had unsuccessfully attempted vaginal delivery elsewhere before being referred to this maternity hospital [29]. Even where cesarean section was indicated by past pregnancy history some women still attempted vaginal delivery until there was a glaring failure with obvious threat to the life of the mother or unborn child. This would also explain the significant association found between fetal distress and SCBU admission underpinned by prolonged/obstructed labor.

Taken together, these factors suggest the need for and the complementary role of community-oriented culturally-appropriate behavior change interventions directed at mothers throughout pregnancy and at delivery besides initiatives to improve the quality of available care in the hospitals [30, 31]. Given the prospects for better intervention outcomes for this group of newborns compared to preterm and low birth weight newborns, improved maternal education as well as enhanced capacity of secondary-level hospitals to effectively manage these factors to curtail the burden of severe illness in the first week of life and the potential long-term sequelae among survivors should be considered as priority. This is consistent with current initiatives to draw global attention to the critical role of functional hospital care for children in the developing world especially against the backdrop of high level of referrals among those born outside hospitals [30].

The findings in this study are likely to be applicable to most hospital settings in resource-poor countries. For example, except for the few university teaching hospitals, many hospitals lack NICU which typically should be furnished with high-tech equipment such as mechanical ventilators, cardio-respiratory monitors and facilities for parenteral nutrition as well as conform to some minimum operational standards [32, 33]. However, a greater number of general or secondary-level hospitals are able to provide a level of service falling short of intensive care but exceeding normal care including incubator care, tube feeding, administering intravenous fluids/drugs, frequent bilirubin estimation and phototherapy [34–36], within the remit of services delivered in SCBU [32]. This study has also highlighted critical service areas within the reach of health authorities in many resource-poor countries for optimizing the survival and growth of full-term infants without prejudice to any opportunity to develop more advanced services to cater for preterm births wherever possible.

Notwithstanding, a number of limitations of this retrospective cohort study are still worth noting. Data on perinatal mortality was not available to determine the survival rates across the various risk factors even though it would have been problematic to assign specific diagnoses to sick newborns without adequate laboratory investigations. However, perinatal mortality among full-term infants is expectedly much lower than among preterm and low birth weight infants who were not considered in this study. For example, infections were commonly documented as sepsis based on clinical suspicion and presumptive diagnosis due to the lack of facilities for blood and/or cerebrospinal fluid culture. It was therefore not possible to determine the most prominent causative pathogens in this study population. Prospective studies addressing these limitations would therefore be worthwhile to establish the full spectrum of service requirements for this group of newborns.

## Conclusion

A significant proportion of newborns requiring special care in resource-poor settings are full-term with normal birth weight. These infants are associated with modifiable risk factors that can be effectively addressed at appropriately equipped secondary-level hospitals to minimize the risk of long-term neuro-developmental conditions in the survivors. Prenatal maternal education is also necessary on the risks facing these apparently viable infants in resource-poor settings.
